# Evaluation of HIF-1 Involvement in the BDNF and ProBDNF Signaling Pathways among Obstructive Sleep Apnea Patients

**DOI:** 10.3390/ijms232314876

**Published:** 2022-11-28

**Authors:** Agata Gabryelska, Marcin Sochal

**Affiliations:** Department of Sleep Medicine and Metabolic Disorders, Medical University of Lodz, 90-419 Lodz, Poland

**Keywords:** OSA, PSG, neurotrophin, hypoxia, oxygen saturation

## Abstract

Obstructive Sleep Apnea (OSA) is a chronic condition characterized by intermittent hypoxia associated with multiple comorbidities, including psychiatric disorders, such as depression, insomnia, and cognitive impairment. The brain-derived neurotrophic factor (BDNF) and proBDNF singling pathways have been shown to be involved in this group of diseases. Furthermore, their expression might be affected by hypoxia-inducible factor 1 (HIF-1), which is an oxygen sensitive transcription factor due to its alpha subunit. Therefore, this study aimed to evaluate the association between HIF-1α, BDNF, and proBDNF protein levels among OSA patients. This study included 40 individuals who underwent polysomnography (PSG) and were divided into the OSA group (*n* = 20; AHI ≥ 30) and healthy control (*n* = 20; AHI < 5) based on the apnea–hypopnea index (AHI). All participants had their peripheral blood collected in the evening before and the morning after the PSG. BDNF, proBDNF, and HIF-1α protein concertation measurements were performed using ELISA. No differences were found in BDNF, proBDNF, and HIF-1α protein levels between OSA and the control group, both in the evening and in the morning. In the OSA group, i.e., the linear regression model, the morning BDNF protein level was predicted by age (ß = −0.389, *p* = 0.023) and the mean SpO2 of desaturations during sleep (ß = −0.577, *p* = 0.002). This model accounted for 63.3% of the variability in the morning BDNF protein level (F = 14.639, *p* < 0.001). The morning proBDNF protein level was predicted by age (ß = −0.395, *p* = 0.033) and HIF-1α morning protein level (ß = −3.192, *p* = 0.005). This model accounted for 52.4% of the variability in the morning BDNF protein level (F = 9.355, *p* = 0.002). The obtained results suggest that the HIF-1 transcription factor might be involved in the pathway activated by proBDNF, which may have protective properties from hypoxia in OSA patients.

## 1. Introduction

Obstructive sleep apnea (OSA) is a chronic condition characterized by recurrent pauses in breathing during sleep caused by the collapse of upper airways. This results in sleep fragmentation due to the presence of frequent arousals, nocturnal desaturations, and excessive daytime sleepiness [1]. The prevalence of the disorder is continuously rising and is estimated to affect between 9% to 38%, with men being at a higher risk [2]. Additionally, studies have suggested that it can exceed 50% in some populations [3]. This increase in OSA diagnosis is associated with the ever-growing obesity presence, as BMI is a key risk factor for the disorder [4,5]. Further, OSA patients are at a high threat of developing many complications, including metabolic [6,7], cardiovascular [8,9], or immunological diseases [10,11,12], as well as psychiatric disorders such as depression [13,14] and insomnia [15,16]. The apneas and hypopneas present during sleep result in recurrent, intermittent hypoxia (IH) at night. The main factor responsible for oxygen metabolism homeostasis is hypoxia-inducible factor 1 (HIF-1), consisting of two subunits, α and β, with the former being oxygen sensitive [17]. Several recent studies have shown the upregulation of HIF-1α protein levels among OSA patients [18,19,20,21]. Furthermore, the HIF-1 act is one of the crucial transcription factors activating a vast number of genes, including the brain-derived neurotrophic factor (BDNF) [22], which is the most widely studied neurotrophin that is responsible for the growth and development of neurons and neuron plasticity. It has been shown that it can cross the blood–brain barrier, making it a focus in studies on the brain and brain-related disorders [23,24]. Before mature BDNFs form, several enzymatic reactions take place, with one of the earlier forms being proBDNF, which, following the split, generates metabolically-active mature BDNF acting through 2 separate signaling pathways—TrkB, which causes apoptosis and p75NTR, which promotes cell survival—and the proBDNF peptide, which acts only on the ladder [23]. Multiple studies have shown that changes in BDNF and proBDNF signaling pathways are present in the development of psychiatric disorders, such as depression [25,26,27], insomnia [28,29], and cognitive impairment [30,31]. Moreover, overexpression of BDNF was observed in diseases associated with an exacerbated inflammation process [32], which is similarly present in OSA [33]. Limited information on BDNF and proBDNF in OSA is available. Therefore, this study aimed to evaluate the association between HIF-1α, BDNF, proBDNF protein levels, and it chose clinical and demographic parameters among OSA patients and compared protein levels between the OSA and the control group.

## 2. Results

Participants were divided into two groups based on the apnea–hypopnea index (AHI). Individuals with AHI < 5 were included in the control group (*n* = 20), while the OSA group (*n* = 20) comprised individuals with AHI ≥ 30. The baseline characteristics of both groups are shown in Table 1.

**Figure 1:**
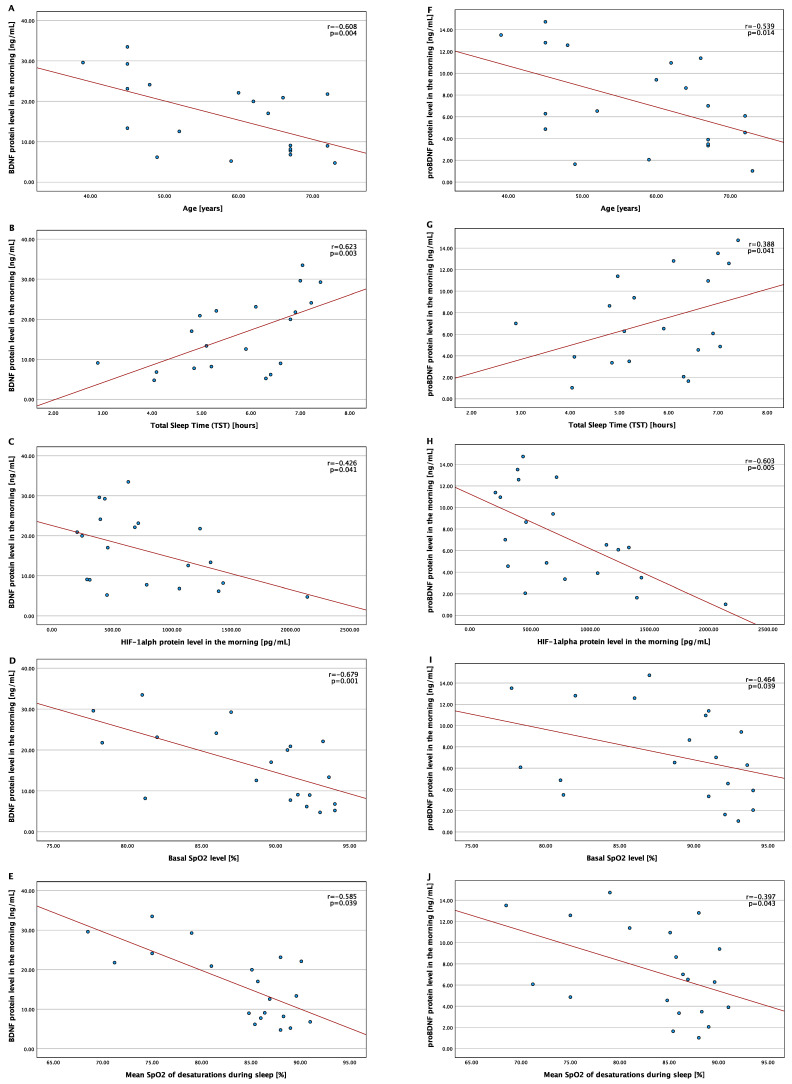
Correlation between morning BDNF and proBDNF protein levels and the chosen variables in the OSA group. Abbreviations: BDNF—brain-derived neurotrophic factor, HIF-1—hypoxia-inducible factor 1, OSA—obstructive sleep apnea, SpO_2_—oxygen saturation. (**A**) Correlation between the BDNF protein level in the morning and age; (**B**) correlation between the BDNF protein level in the morning and total sleep time (TST); (**C**) correlation between the BDNF protein level in the morning and HIF-1 alpha protein level in the morning; (**D**) correlation between the BDNF protein level in the morning and Basal SpO_2_ level; (**E**) correlation between the BDNF protein level in the morning and mean SpO_2_ of desaturations during sleep; (**F**) correlation between the proBDNF protein level in the morning and age; (**G**) correlation between the proBDNF protein level in the morning and total sleep time (TST); (**H**) correlation between the proBDNF protein level in the morning and HIF-1 alpha protein level in the morning; (**I**) correlation between the proBDNF protein level in the morning and Basal SpO_2_ level; (**J**) correlation between the proBDNF protein level in the morning and mean SpO_2_ of desaturations during sleep.

In the OSA group, morning BDNF and proBDNF protein levels correlated with age (r = −0.608, *p* = 0.004 and r = −0.539, *p* = 0.014, respectively), morning HIF-1α protein level (r = −0.426, *p* = 0.041 and r = −0.603, *p* = 0.005, respectively), TST (r = 0.623, *p* = 0.003, and r = 0.388, *p* = 0.041, respectively), basal SpO2 level (r = −0.679, *p* = 0.001 and r = −0.464, *p* = 0.039, respectively), as well as the mean SpO2 of desaturations during sleep (r = −0.585, *p* = 0.039 and r = −0.397, *p* = 0.043, respectively). The correlations are shown in Figure 1. No statistically significant associations were observed between morning protein levels and BMI, AHI, and arousal index.

In the linear regression model in OSA patients, the morning BDNF protein level was predicted by age (ß = −0.389, *p* = 0.023) and mean SpO_2_ of desaturations during sleep (ß = −0.577, *p* = 0.002). This model accounted for 63.3% of the variability in the morning BDNF protein level (F = 14.639, *p* < 0.001). The morning proBDNF protein level was predicted by age (ß = −0.395, *p* = 0.033) and the HIF-1α morning protein level (ß = −3.192, *p* = 0.005). This model accounted for 52.4% of the variability in the morning BDNF protein level (F = 9.355, *p* = 0.002). The full results of the regression models are shown in Table 2.

## 3. Discussion

Multiple studies have shown the association between the dysregulation of the BDNF signaling pathway and the development and progression of depression and cognitive decline [25,26,27,30,31], both of which are frequently diagnosed comorbidities in OSA patients. In this study, we did not observe differences between healthy individuals and OSA patients in the BDNF and proBDNF protein levels. Further, the groups were not age-matched, which should be taken into consideration. Regardless the available data on differences in the BDNF levels in similar comparisons in the literature is inconclusive, with the majority of studies similarly not noting any changes in the level of this protein [34,35,36,37,38]. In opposition are the results obtained by Flores et al., who, in their study, observed an increased level of the BDNF serum protein level in OSA patients compared to the control group [39]. Further, they showed that the BDNF protein level correlated with individuals’ cognitive performance. What is quite interesting and clinically promising is the continuous positive airway pressure (CPAP) treatment, which reversed this trend. In a similar vein was the data presented by Arslan et al., who observed differences in the BDNF protein levels between the control, mild OSA, and moderate-to-severe OSA. However, no post-hoc examination was performed to evaluate between which groups the differences were present. Additionally, no correlations between the concentration of this neurotrophin and the disorders’ severity were observed [40].

Further, in the OSA group, we achieved an association between the concentration of the studied neurotrophin levels and clinical hypoxia parameters, biochemical parameters in the HIF-1α protein level, total sleep time, and age. Notably, the HIF-1α protein level was a significant predictor of proBDNF, but not the BDNF protein level. This observation is particularly interesting considering the fact that proBDNF acts only through the p75NTR, which promotes cell survival [23,41]. This might suggest that this pathway is activated through the HIF-1 transcription factor, which in earlier studies was shown to be increased in OSA patients [18,20]. In this study, we did not observe such an overexpression of HIF-1α, however it might be due, yet again, to a small study sample, which is one of the main limitations of the study, and assumptions made in the study should be further evaluated in a larger group. The results of the linear regression may further indicate the possible activation of proBDNF in a HIF-1 manner, which is not present in BDNF. Thus, proBDNF might act as a protective defense during hypoxia present in OSA during the night. It is vital to highlight that, in the present study, we did not evaluate the cognitive performance of included participants, and this can also influence the ambiguity of the achieved results.

Furthermore, age was a strong predictive factor of both BDNF and proBDNF protein levels, which is in line with the available data. Mizoguzi et al. have shown that the BDNF level decreases with age and cognitive impairment [31]. Several studies similarly imply comparable dependencies for the proBDNF protein [42,43,44]. Based on these data, it seems that age is a key parameter associated with the BDNF and proBDNF protein levels, even in OSA patients, possibly meaning hypoxia is as a secondary parameter that influences the BDNF and proBDNF.

## 4. Materials and Methods

### 4.1. Sample

The study group included 40 individuals who were referred to the Sleep and Respiratory Disorders Center in Łódź (Poland) with a presumptive diagnosis of OSA. All participants underwent standard nocturnal polysomnography examination. The exclusion criteria of the study consisted of the diagnosis of chronic respiratory disorders (such as bronchial asthma, chronic obstructive pulmonary disease, or any chronic pulmonary disorder resulting in respiratory failure), chronic inflammatory diseases (such as lupus or inflammatory bowel disease), psychiatry disorders (such as schizophrenia, bipolar disorder, or depression), cancer (active or in medical history), and pregnancy. Additionally, individuals with infection within 1 month of blood collection, an international flight 2 weeks prior to the sleep laboratory examination, and usage of medication known to affect sleep during the 2 weeks before or during the sleep laboratory examination. The study was approved by the Ethics Committee of the Medical University of Lodz (RNN/432/18/KE; 10 December 2018). All patients provided written informed consent to participate in the study.

### 4.2. Polysomnography

Patients were admitted to the sleep lab at 21:00 h (±0.5 h) and underwent physical examination (measurement of body mass, height, heart rate, and blood pressure). Nocturnal polysomnography was performed by recording the following channels: Electroencephalography (C4/A1, C3/A2), chin muscles and anterior tibialis electromyography, electrooculography, measurements of oro-nasal airflow (a thermistor gauge), snoring, body position, respiratory movements of chest and abdomen (piezoelectric gauges), unipolar electrocardiogram and haemoglobin oxygen saturation (SpO_2_) (Alice 6, Philips-Respironics, Murrysville, PA, USA). Sleep stages were scored according to the criteria based on a 30 s epoch standard [45]. Apnea was attained with the reduction of airflow to less than 10% of the baseline for at least 10 s. Hypopnea was defined as at least a 30% reduction of air flow for at least 10 s, accompanied by an over 3% decrease in SpO_2_ or arousal. Encephalography arousals were scored according to the American Academy of Sleep Medicine guidelines [45].

### 4.3. Material Collection and Protein Level Assessment

Peripheral blood samples were collected in the evening (at 21:00–21:30, 15 min before lights out) before and in the morning (at 6:00–6:30, 15 min after light on), following PSG examination, and centrifuged. The serum was collected and stored at −80 °C. Enzyme-linked immunosorbent assay kits were used to assess HIF-1α (Invitrogen, Carlsbad, CA, USA), BDNF (FineTest, Wuhan, China), and proBDNF (FineTest, Wuhan, China) serum levels. The absorbance was measured at a λ = 450 nm wavelength by the absorbance reader (BioTek 800 TS, Agilent Technologies, Santa Clara, CA, USA).

### 4.4. Statistical Analysis

Statistical analysis was performed with SPSS 28.0 (IBM, Armonk, NY, USA). Normal distribution was evaluated using the Shapiro-Wilk test. Comparisons between the groups for data with normal distribution were performed by independent-samples t-test, and the results were presented as mean ± standard deviation, while for data without a normal distribution, a Mann-Whitney U test was used, and the results were presented as the median and interquartile range (IQR). The chi^2^ test was used for categorical variables. For repeated dependent measurements (evening-morning protein concentration differences), paired sample Wilcoxon tests were used for data with non-normal distribution. Correlations were determined using Spearman’s rank correlation. Linear regression was performed to analyze the effect of age, total sleep time (TST), HIF-1α morning protein level, basal SpO_2_, and mean SpO_2_ of desaturations during sleep on the morning BDNF and proBDNF protein level in OSA patients. The level of significance was set at *p* < 0.05.

## 5. Conclusions

In the present study, we did not observe differences in the BDNF and proBDNF protein levels between healthy participants and OSA patients. However, in the OSA group, the level of proBDNF was significantly predicted by the level of HIF-1α, while for BDNF, only basal SpO_2_ was a risk factor from all hypoxia parameters, suggesting that the HIF-1 transcription factor might be involved in the protective pathway associated with the proBDNF peptide. Regardless, age is an important factor associated with evaluated neurotrophins. More studies are needed to fully understand the relationship between hypoxia present in OSA patients and neurotrophin expression. Future research should include cognitive assessment as well as OSA patients with comorbid depression and insomnia to better understand the mechanism responsible for the development of these OSA comorbidities and the involvement of neurotrophins in this process.

## Figures and Tables

**Table 1:** Baseline characteristics of the study groups.

		Control Group (*n* = 20)	OSA Group (*n* = 20)	*p*-Value
**Demogra-phic data**	Sex [M(%)/F(%)]	11(55%)/9(45%)	17(85%)/3(15%)	0.019
	Age [years]	46.00 ± 13.30	61.00 ± 11.04	0.009
	BMI [kg/m^2^]	26.00 (24.08–29.39)	37.49 (32.94–40.74)	<0.001
**Polysomnography**	Total Sleep Time (TST) [h]	5.97 ± 0.98	5.75 ± 1.24	0.531
	Arousal index [events/h]	9.80 (6.20–16.63)	29.25 (21.5–38.20)	<0.001
	AHI [events/h]	1.45 (1.08–2.50)	58.00 (48.40–76.13)	<0.001
	Total number of desaturations	8.00 ± 5.94	325.5 ± 210.15	<0.001
	Desaturation Index	1.5 ± 0.97	63.2 ± 24.72	<0.001
	Basal SpO_2_ level	94.20 (93.00–95.10)	90.90 (83.00–92.83)	<0.001
	Mean SpO_2_ of desaturations during sleep	91.00 (90.05–92.68)	85.85 (79.50–88.23)	<0.001
**Protein Concentration**	BDNF evening [ng/mL]	14.12 (6.82–23.27)	9.79 (6.08–24.56)	0.417
	BDNF morning [ng/mL]	15.79 ± 8.22	16.21 ± 9.07	0.858
	proBDNF evening [ng/mL]	7.75 (3.49–10.11)	4.74 (2.54–11.21)	0.579
	proBDNF morning [ng/mL]	7.12 ± 4.32	7.24 ± 4.26	0.930
	HIF-1α evening [pg/mL]	646.21 (317.83–1031.25)	551.13 (286.17–1103.04)	0.656
	HIF-1α morning [pg/mL]	706.16 (460.33–1226.25)	660.00 (390.33–1212.51)	0.449

Abbreviations: BDNF—brain-derived neurotrophic factor, BMI—body mass index, F—female, HIF-1—hypoxia-inducible factor 1, M—male, OSA—obstructive sleep apnea, SpO_2_—oxygen saturation.

**Table 2:** Linear regression models for the morning BDNF and proBDNF protein levels in OSA patients.

Parameters Included in the Linear Regression	BDNF Morning Protein Level			ProBDNF Morning Protein Level		
	(r^2^ = 0.633, F = 14.639, *p* < 0.001)			(r^2^ = 0.524, F = 9.355, *p* = 0.002)		
	B	t	*p*	B	t	*p*
Age	−0.389	−2.496	0.023	−0.395	−2.327	0.033
Total Sleep Time (TST)	0.073	0.342	0.737	0.031	0.149	0.883
HIF-1α morning protein level	−0.246	−1.678	0.113	−0.542	−3.192	0.005
Basal SpO_2_	−0.164	−0.741	0.470	−0.237	−1.363	0.192
Mean SpO_2_ of desaturations during sleep	−0.577	−3.707	0.002	−0.183	−0.999	0.333

Abbreviations: BDNF—brain-derived neurotrophic factor, HIF-1—hypoxia-inducible factor 1, OSA—obstructive sleep apnea, SpO_2_—oxygen saturation.

## Data Availability

Data will be made available upon request.
